# SARS-Co-V2 infection in never, former, and current tobacco/nicotine users: a cohort study of 4040 Egyptian healthcare workers

**DOI:** 10.1186/s12889-021-11290-x

**Published:** 2021-06-28

**Authors:** Aya Mostafa, Manal H. El-Sayed, Mahmoud El-Meteini, Ayman Saleh, Ashraf Omar, Ossama Mansour, Samia Girgis, Hala Hafez, Sahar Kandil

**Affiliations:** 1 Department of Community, Environmental, and Occupational Medicine, Faculty of Medicine, Ain Shams University, 38 Ramses st., Abbassia square, PO-box 11566, Cairo, Egypt; 2 Department of Pediatrics, Faculty of Medicine, Ain Shams University, Cairo, Egypt; 3 Clinical Research Center (MASRI-CRC), Faculty of Medicine, Ain Shams University, Cairo, Egypt; 4 Department of Hepatobiliary Surgery & Liver Transplantation, Ain Shams Center for Organ Transplantation (ASCOT), Faculty of Medicine, Ain Shams University, Cairo, Egypt; 5 Department of Cardiology, Faculty of Medicine, Ain Shams University, Cairo, Egypt; 6 Department of General Surgery, Faculty of Medicine, Ain Shams University, Cairo, Egypt; 7 Department of Otolaryngology, Head and Neck Surgery, Faculty of Medicine, Ain Shams University, Cairo, Egypt; 8 Department of Clinical Pathology, Faculty of Medicine, Ain Shams University, Cairo, Egypt

**Keywords:** Tobacco, Nicotine, Smoking, COVID-19, SARS-co-V2, Cohort, Healthcare workers, Egypt

## Abstract

**Background:**

Smoking negatively impacts COVID-19 severity and adverse outcomes. Evidence on whether smoking is associated with SARS-Co-V2 infection and having a positive test is scarce, particularly from low-and middle-income countries, where most of the world’s billion smokers live. The inconsistency in relevant findings calls for study designs and analyses to account for possible confounders including background characteristics and pre-existing co-morbidities, to disentangle the specific effect of smoking. In healthcare workers (HCWs) the frequency of exposure to COVID-19 cases adds another layer of risk that was not factored in previous studies. We examined the association of HCWs’ tobacco/nicotine use (never, former, and current use) with having a positive SARS-Co-V2 test result and symptoms suggestive of infection, accounting for demographics, exposures, and co-morbidities.

**Methods:**

A prospective cohort study of 4040 healthcare workers with baseline and follow-up screening took place during April–June 2020 in 12 healthcare facilities in Cairo, Egypt. Data on demographics, tobacco/nicotine use (manufactured or roll-your-own cigarettes, waterpipe tobacco, and electronic devices), co-morbidities, symptoms, exposures, and SARS-Co-V2 investigations were analyzed. Multinomial and multivariable logistic regression analyses were performed.

**Results:**

Overall, 270/4040 (6.7, 95%CI: 5.9–7.5) had positive SARS-CoV-2 tests, 479 (11.9%) were current and 79 (2.0%) were former tobacco/nicotine users. The proportion of positive tests was 7.0% (243/3482, 95%CI: 6.1–7.8) among never, 5.1% (4/79, 95%CI: 0.1–10.0) among former, and 4.8% (23/479, 95%CI: 2.9–6.7) among current users. HCWs’ SARS-CoV-2 test results did not vary significantly by single/multiple or daily/non-daily tobacco/nicotine use. Compared to never users, former users were more likely to self-report a pre-existing medical condition (OR_adjusted_1.87, 95%CI: 1.05–3.33, *p* = 0.033), and to experience symptoms suggestive of COVID-19 (OR_adjusted_1.76, 95%CI: 1.07–2.90, *p* = 0.027). After adjustment, former (OR_adjusted_0.45, 95%CI: 0.11–1.89, *p* = 0.273) and current (OR_adjusted_0.65, 95%CI: 0.38–1.09, *p* = 0.101) tobacco/nicotine use was not associated with HCWs’ SARS-CoV-2 positive test results.

**Conclusions:**

This is the first report on this association from low- and middle-income countries with high tobacco/nicotine use prevalence. In this HCW cohort, having a positive SARS-CoV-2 test was not associated with tobacco/nicotine use after accounting for demographics, exposures, and co-morbidities. Additional population-based studies could use such preliminary evidence to investigate this controversial association.

**Supplementary Information:**

The online version contains supplementary material available at 10.1186/s12889-021-11290-x.

## Introduction

Tobacco use is a deadly habit that kills over 8 million individuals annually and is the sole preventable risk factor for the major non-communicable diseases that contribute to 80% of premature mortality worldwide [1]. Smoking increases the risk for viral and bacterial respiratory infections [2]. Since the beginning of the COVID-19 pandemic, a growing body of literature has examined whether smokers are at increased risk of symptomatic or severe COVID-19 and its complications [3]. However, evidence on whether smoking is associated with SARS-Co-V2 infection and having a positive test is scarce, particularly from low-and-middle income countries (LMICs), where most of the world’s billion smokers live [4].

A meta-analysis of 233 studies revealed that current smokers showed a reduced risk of SARS-Co-V2 infection, while former smokers showed an increased risk of severe COVID-19 and its complications [3]. However, the authors could not conclude causality [3]. Some reports indicate current smoking rates in hospitalized COVID-19 patients were lower than in the general population [5]. On the other hand, current smoking was independently associated with self-reported confirmed COVID-19 in 53,002 adults in the UK [6]. Findings from a cohort of 2.4 million people in the UK also suggest current smoking is associated with symptomatic COVID-19 [7].

The inconsistency in findings about the role of smoking in COVID-19 so far initiated the call for study designs and analyses to take into consideration the interplay between participants’ smoking status, background characteristics, and pre-existing co-morbidities (and representativeness of these data for the general population) to unravel the specific effect of smoking [8]. In healthcare workers (HCWs), the frequency of exposure to COVID-19 cases may add another layer of risk that was not factored in previous studies.

In Egypt, the first confirmed case of COVID-19 was reported in mid-February 2020. By mid-March 2021, a total of 193,482 COVID-19 cases and 11,472 deaths had been reported (~ 3.5% of cases and deaths were HCWs) [9]. Current smoking rates among Egyptian HCWs are lower than (8.0%) or equal to (22.7%) those in the general population [10, 11]. In the 2017 national STEPS survey, it was estimated that 3.2% (6.1% males and 0.1% females) of Egyptians aged 15–69 years were former smokers and 22.7% (43.4% males and 0.5% females) were current smokers. Among this population, 18.6% smoked cigarettes (35.9% males and 0.3% females) and 4.5% smoked waterpipe tobacco (8.7% males and 0.1% females) [12]. Also, 0.6% used electronic cigarettes (1.2% males and 0.1% females) [12]. The national prevalence rates of hypertension, diabetes, obesity, and chronic obstructive pulmonary diseases (COPD) were 29.5, 15.5, 35.7, and 3.5%, respectively [12, 13].

A prospective investigation including baseline and follow-up screening and risk assessment of HCWs (SARAH: NCT04348214) took place between 21 April and 10 June 2020 in 12 Ain Shams University (ASU) healthcare facilities in Cairo, Egypt to measure the extent of SARS-CoV-2 infection among 4040 HCWs [14, 15]. The current study examined the association of HCWs’ tobacco/nicotine use status (never, former, and current use) with having a positive SARS-Co-V2 test result at baseline or follow-up, accounting for background characteristics, exposures and co-morbidities. We also assessed whether symptoms suggestive of COVID-19 (and the severity thereof) varied by tobacco/nicotine use status. In addition, we explored if former users had quit due to the health warnings about the association of tobacco use with COVID-19.

## Methods

### Study design and participants

A prospective cohort study took place between 21 April and 10 June 2020 in 12 ASU healthcare facilities. The study consisted of two phases including baseline screening and follow-up, 21 days apart, in accordance with the World Health Organization’s sero-epidemiological investigation protocol for COVID-19 available at that time [16]. The baseline and follow-up study design, setting, participants, tools, procedures, and findings have been previously described [14, 15]. Each phase included an online survey to assess healthcare workers’ background, exposure, and clinical data plus laboratory testing using RT-PCR and rapid serological assays to assess SARS-CoV-2 infection.

All on-job HCWs were invited to participate voluntarily at the baseline screening phase with no exclusion criteria. HCWs provided a written informed consent during the baseline recruitment process. The same HCWs who participated at baseline were invited to participate in the follow-up phase. This study was approved by the Ethics Review Committee, Faculty of Medicine, ASU (FMASUP18b/2020).

### Data collection

At each of the 12 healthcare facilities, HCWs visited workstations dedicated for baseline and follow-up screening procedures. At one workstation, HCWs completed the online survey using a unique study ID for anonymous linkage with laboratory results. At the second workstation, laboratory samples including a 5-ml venous blood sample and a combined nasal and oropharyngeal swabs were collected. Detailed methodology has been previously described [14, 15].

### Study tools

Details of the online survey development and laboratory tests have been previously described [14]. The baseline survey included questions on demographic characteristics, symptoms and pre-existing medical conditions and contact with a COVID-19 case. The follow-up survey included questions on exposures and symptoms that occurred in the period since baseline screening. SARS-CoV-2 IgM and IgG antibodies were detected using the lateral flow immunochromatographic assay Artron® One Step COVID-19 IgM/IgG Antibody Test (Artron Laboratories Inc., Canada) [17, 18]. The assay has an estimated sensitivity and specificity of 83.3 and 100%, respectively by Lassaunière et al. [17] and 94.4 and 98.2% respectively by Zhang and Zheng [18]. RT-PCR testing was done for all HCWs at baseline and for seropositive cases only at follow-up using *CerTest* Viasure® SARS-CoV-2 Real Time PCR Detection Kit (CerTest, Biotec, Spain) [14, 19]. The assay has 97.5% sensitivity and > 99.9% specificity [20].

### Measures

The measures included from baseline and follow-up data in the current study are HCWs’ demographic characteristics, pre-existing medical conditions, tobacco use, symptoms and symptoms severity, exposure to a confirmed COVID-19 case, and SARS-Co-V2 test results.

#### Positive SARS-co-V2 test

For the current study, HCWs were considered to have a positive test result (*n* = 270) if any of the three tests (IgM, IgG, and PCR) were positive at baseline (*n* = 170/4040) or follow-up (*n* = 100/2282). If all the three test results were negative, the HCW was considered to have a negative test result.

#### Demographic characteristics

HCWs were asked at baseline about their age (in years), gender, governorate of residence, urban/rural residence, educational attainment, marital status, and occupation (physician, nurse, non-clinical care).

#### Self-reported pre-existing medical conditions

HCWs self-reported at baseline whether they had any of the following condition(s): obesity, diabetes, heart disease, COPD (requiring medication/not), chronic liver/blood/kidney/neurological diseases, cancer, immune deficiency, organ or bone marrow transplant receipt, or pregnancy.

#### Tobacco/nicotine use

HCWs were asked at baseline about their past 30-day use of any of the following tobacco/nicotine products: manufactured or roll-your-own cigarettes, waterpipe tobacco, and electronic devices. If the response was positive, the HCW was considered a “current user” and was asked about current use frequency (daily/non-daily). If HCWs were not current users, they were asked whether they had used any tobacco/nicotine product in the past. The HCW who used tobacco/nicotine in the past was considered a “former user” and was asked about the time of quitting (this month, a month ago, 2–5 months ago, 6–11 months ago, ≥ a year ago) and if quitting was due to health warnings about the association between smoking or tobacco use with COVID-19. If HCWs were not a “current” or “former” user, they were considered “never” users. Current users of more than one product were considered “multiple users”; no product was given a priority over the other, and users of only one product were considered “single users”.

#### Symptoms and symptoms severity

HCWs were provided a list of symptoms suggestive of COVID-19 at baseline and follow-up and were asked to report the occurrence and type of symptoms: fever < 38 °C, fever ≥38 °C, chills, fatigue, muscle pain, joint pain, sore throat, dry cough, cough with sputum, runny nose/nasal congestion, sneezing, shortness of breath, wheezing, chest pain, other respiratory symptoms, nausea, vomiting, abdominal discomfort/pain, diarrhea, headache, confusion, loss of appetite, change/loss of taste or smell, skin rash and conjunctivitis. HCWs reporting any symptoms were asked to rate their symptoms as mild, moderate, or severe. They were also asked whether they have been hospitalized at the baseline and follow-up surveys.

#### Contact with a confirmed COVID-19 case

HCWs reported if they have been exposed to a confirmed COVID-19 case (i.e. confirmed through RT-PCR laboratory testing) at baseline and follow-up.

### Statistical analysis

We tested for associations between tobacco/nicotine use, exposure, clinical, and demographic characteristics, and the HCWs’ SARS-Co-V2 positive test result using the Chi-squared test or Fisher’s Exact test, and binary logistic regression analysis. Unadjusted odds ratios (OR) and 95% confidence intervals (CI) are reported. Multinomial logistic regression analyses were conducted to examine factors associated with HCWs’ tobacco/nicotine use status as the dependent variable (never user (referent), former and current users). Three separate multivariable logistic regression models (overall, at baseline, and at follow-up) were conducted with the dependent variable being the HCWs’ SARS-Co-V2 positive test results to examine whether it was independently associated with tobacco/nicotine use status (never user (referent), former and current users)—adjusting for demographic characteristics, presence of pre-existing medical conditions, presence of symptoms, and contact with a confirmed COVID-19 case. To account for a hypothesized differential effect of tobacco/nicotine use on SARS-Co-V2 positivity according to the presence of pre-existing medical conditions, an interaction term between tobacco/nicotine use and pre-existing medical conditions was introduced into the models. In addition to including the total sample, regression was conducted excluding electronic device users to rule out any putative protective effect of nicotine delivered with their use. We reported adjusted OR (OR_adjusted_) and 95% CIs. Effect estimates and *p*-values are provided. SPSS version 25 was used for all analyses.

## Results

### Demographic characteristics and pre-existing medical conditions

Background characteristics of the 4040 HCWs are presented in Table 1. Most HCWs attained high levels of education: 52.5% (2122/4040) attained university or higher and 38.4% (1551/4040) attained secondary or equivalent education. In total, 17.4% (701/4040) of HCWs self-reported having a pre-existing medical condition (Table 2); the most common were hypertension (*n* = 291, 7.2%), diabetes (*n* = 176, 4.4%), obesity (*n* = 145, 3.6%), COPD (*n* = 95, 2.4%), and cardiovascular diseases (*n* = 55, 1.4%) (Supplementary Table 1). Former tobacco/nicotine users were significantly more likely to self-report a pre-existing medical condition (19/79, 24.1%) than never (623/3482, 17.9%) and current (59/479, 12.3%) users (Table 2), particularly obesity, cardiovascular diseases and COPD (Supplementary Table 1).

**Table 1:** Demographic characteristics of healthcare workers by tobacco/nicotine use status (*n* = 4040)

	Tobacco/nicotine use status				χ2	p-value^a^
	Total	Never	Former	Current		
	N = 4040	***n*** = 3482	***n*** = 79	***n*** = 479		
	n (%)	n (%)	n (%)	n (%)		
**Age**						
18–24	603 (14.9)	510 (14.6)	11 (13.9)	82 (17.1)	1.646	0.200
25–29	1279 (31.7)	1117 (32.1)	23 (29.1)	139 (29.0)		
30–39	1057 (26.2)	892 (25.6)	22 (27.8)	143 (29.9)		
40–49	700 (17.3)	607 (17.4)	15 (19.0)	78 (16.3)		
≥ 50	401 (9.9)	356 (10.2)	8 (10.1)	37 (7.7)		
**Gender**						
Female	2486 (61.5)	2456 (70.5)	8 (10.1)	22 (4.6)	839.302	< 0.001
Male	1554 (38.5)	1026 (29.5)	71 (89.9)	457 (95.4)		
**Governorate of residence**						
Cairo	3062 (75.8)	2714 (77.9)	55 (69.6)	292 (61.0)	67.789	< 0.001
Outside Cairo	978 (24.2)	768 (22.1)	24 (30.4)	187 (39.0)		
**Urban/rural residence**						
Urban	3543 (87.7)	3119 (89.6)	64 (81.0)	360 (75.2)	84.328	< 0.001
Rural	497 (12.3)	363 (10.4)	15 (19.0)	119 (24.8)		
**Marital status**						
Not married	1865 (46.2)	1629 (46.8)	37 (46.8)	199 (41.5)	4.446	0.035
Married	2175 (53.8)	1853 (53.2)	42 (53.2)	280 (58.5)		
**Education**						
University/higher	2122 (52.5)	1912 (54.9)	41 (51.9)	169 (35.3)	83.848	< 0.001
Secondary/equivalent	1551 (38.4)	1307 (37.5)	27 (34.2)	217 (45.3)		
Primary/preparatory	238 (5.9)	159 (4.6)	8 (10.1)	71 (14.8)		
Less than primary	129 (3.2)	104 (3.0)	3 (3.8)	22 (4.6)		
**Occupation**						
Physician	1577 (39.0)	1433 (41.2)	34 (43.0)	110 (23.0)	10.528	0.001
Nurse	1598 (39.6)	1398 (40.1)	21 (26.6)	179 (37.4)		
Non-clinical care	865 (21.4)	651 (18.7)	24 (30.4)	190 (39.7)		
**Returned for Follow up**	2355 (58.3)	2049 (58.8)	42 (53.2)	264 (55.1)	3.282	0.194

^a^ Chi-squared test or Fisher’s Exact test

**Table 2:** Exposure history and clinical characteristics of healthcare workers by tobacco/nicotine use status (n = 4040)

		Tobacco/nicotine use status			χ2	*p*-value^a^
	Total	Never	Former	Current		
	*N* = 4040	*n* = 3482	*n* = 79	*n* = 479		
	*n* (%)	*n* (%)	*n* (%)	*n* (%)		
**Pre-existing medical condition, yes**	701 (17.4)	623 (17.9)	19 (24.1)	59 (12.3)	7.433	0.006
**Contact with a confirmed case, yes**^b^	1558 (38.6)	1361 (39.1)	31 (39.2)	166 (34.7)	3.505	0.173
**Symptoms, yes**^b^	1224 (30.3)	1088 (31.2)	27 (34.2)	109 (22.8)	14.949	0.001
**Hospitalized, yes**^b^	120 (3.0)	102 (2.9)	2 (2.5)	16 (3.3)	0.301	0.860
**Symptoms severity**^b^	***n*** **= 1224**	***n*** **= 1088**	***n* = 27**	***n*** **= 109**		
Mild	652 (53.3)	575 (52.8)	17 (63.0)	60 (55.0)	1.728	0.786
Moderate	459 (37.5)	411 (37.8)	9 (33.3)	39 (35.8)		
Severe	113 (9.2)	102 (9.4)	1 (3.7)	10 (9.2)		
**SARS-Co-V2 test result**						
Negative	3770 (93.3)	3239 (93.0)	75 (94.9)	456 (95.2)	3.410	0.065
Positive at baseline	170 (4.2)	152 (4.4)	3 (3.8)	15 (3.1)		
Positive at follow-up	100 (2.5)	91 (2.6)	1 (1.3)	8 (1.7)		
**Among total SARS-CoV-2 positive**	***n*** **= 270**	***n*** **= 243**	***n* = 4**	***n* = 23**		
**Symptoms, yes**^b^	109 (40.4)	100 (41.2)	2 (50.0)	7 (30.4)	1.159	0.560
**Symptoms severity**^b^	***n* = 109**	***n* = 100**	***n* = 2**	***n* = 7**		
Mild	40 (36.7)	36 (36.0)	1 (50.0)	3 (42.9)	1.508	0.911
Moderate	55 (50.5)	50 (50.0)	1 (50.0)	4 (57.1)		
Severe	14 (12.8)	14 (14.0)	0 (0.0)	0 (0.0)		

^a^ Chi-squared test or Fisher’s Exact test

^b^ Numbers indicate any occurrence at either baseline or follow-up phases

### Positive SARS-CoV-2 tests

Overall, 6.7% (270/4040; 95%CI: 5.9–7.5) of HCWs had a positive SARS-CoV-2 test between 21 April and 10 June 2020. A total of 4040 HCWs participated in the baseline screening phase, of whom 170 (4.2%) tested positive for either IgM, IgG, or PCR. A total of 2355/4040 (58.3%) returned for follow-up. Another 100/4040 (2.5%) HCWs tested positive for one of the three tests at follow-up (Table 2).

### Tobacco/nicotine use

Out of the 4040 HCWs, 479 (11.9%) were current and 79 (2.0%) were former tobacco/nicotine users. Current users mostly smoked cigarettes (440/479, 91.9%); a fifth (96/479) smoked waterpipe tobacco, 3.9% (19/479) smoked roll-your-own cigarettes, and 7.1% (34/479) used an electronic device. Most current users used a single tobacco/nicotine product (400/479, 83.5%) and were daily users (387/479, 80.8%) (Table 3). Former users who had a positive SARS-CoV-2 test result (*n* = 4) had quit a month or 2–5 months prior to baseline screening, three (75.0%) of whom reported they had quit due to the health warnings about the association of tobacco use with COVID-19 (Table 3).

**Table 3:** Tobacco/nicotine use profile in healthcare workers and SARS-Co-V2 test result (n = 4040)

	Total	SARS-Co-V2 test		χ2	***p***-value^a^	Proportion of positive test
		Negative	Positive			
	N = 4040	***n*** = 3770	n = 270			Overall = 6.7 (5.9–7.5)
	n (%)	n (%)	n (%)			row % (95% CI) i.e. SARS-Co-V2 positive test/Total
**Tobacco/nicotine use status**						
Never	3482 (86.1)	3239 (58.9)	243 (90.0)	3.532	0.060	7.0 (6.1–7.8)
Former	79 (2.0)	75 (2.3)	4 (1.6)	0.439	0.508	5.1 (0.1–10.0)
Current	479 (11.9)	456 (12.1)	23 (8.5)	3.085	0.079	4.8 (2.9–6.7)
**Current users**						
**Number of product use**	**N = 479**	***n*** **= 456**	**n = 23**			
Single	400 (83.5)	380 (83.3)	20 (87.0)	0.209	1.000	5.0 (2.9–7.2)
Multiple	79 (16.5)	76 (16.7)	3 (13.0)			3.8 (−0.5–8.1)
**Daily current use**						
Non-daily	92 (19.2)	88 (19.3)	4 (17.4)	0.051	1.000	4.4 (0.1–8.6)
Daily	387 (80.8)	368 (80.7)	19 (82.6)			4.9 (2.8–7.1)
**Type and frequency of current use**^b^						
Cigarettes	440 (91.9)	418 (91.7)	22 (95.7)	0.465	1.000	5.0 (2.9–7.0)
Daily use, n	366	348	18			
Waterpipe	96 (20.0)	93 (20.4)	3 (13.0)	0.738	0.593	3.1 (−0.4–6.7)
Daily use, n	70	67	3			
Roll your own	19 (3.9)	19 (4.2)	0	0.998	1.000	0
Daily use, n	16	16	0			
Electronic device	34 (7.1)	33 (7.2)	1 (4.3)	0.277	1.000	2.9 (−0.3–8.9)
Daily use, n	27	27	0			
**Former users**						
**Time of quitting**	**n = 79**	***n*** **= 75**	**n = 4**			
This month	4 (5.1)	4 (5.3)	0	5.040	0.197	0
A month ago	13 (16.4)	11 (14.7)	2 (50.0)			15.4 (−7.3–38.1)
2–5 months ago	24 (30.4)	22 (29.3)	2 (50.0)			8.3 (−3.6–20.3)
6–11 months ago	8 (10.1)	8 (10.7)	0			0
A year or more ago	30 (38.0)	30 (40.0)	0			0
**Quit due to health warnings about the association of tobacco use with COVID-19**						
No	58 (73.4)	57 (76.0)	1 (25.0)	5.061	0.055	1.7 (−1.7–5.2)
Yes	21 (26.6)	18 (24.0)	3 (75.0)			14.3 (−0.2–30.6)
**Time of quitting by symptoms**		**No symptoms**	**Symptoms**			
	**n = 79**	***n*** **= 52**	**n = 27**			
This month	4 (5.1)	4 (7.7)	0 (0.0)	2.831	0.606	
A month ago	13 (16.4)	9 (17.3)	4 (14.8)			
2–5 months ago	24 (30.4)	16 (30.8)	8 (29.6)			
6–11 months ago	8 (10.1)	4 (7.7)	4 (14.8)			
A year or more ago	30 (38.0)	19 (36.5)	11 (40.7)			

^a^ Chi-squared test or Fisher’s Exact test

^b^ More than one option was allowed, therefore, numbers and percentages do not add to the total of current use

### SARS-CoV-2 test results by tobacco/nicotine use status

Of the 270 HCWs who had positive SARS-Co-V2 tests, 10.0% were former (n = 4, 1.5%) and current (*n* = 23, 8.5%) tobacco/nicotine users (Table 3). The proportion of positive SARS-CoV-2 tests was 7.0% (243/3482, 95%CI: 6.1–7.8) among never users, 5.1% (4/79, 95%CI: 0.1–10.0) among former users, and 4.8% (23/479, 95%CI: 2.9–6.7) among current users. HCWs’ SARS-CoV-2 test results did not differ significantly by tobacco/nicotine use status, single/multiple or daily/non-daily use (Table 3).

### Symptoms by tobacco/nicotine use status

Overall, 30.3% (1224/4040) of HCWs reported symptoms suggestive of COVID-19 at baseline or follow-up; more likely among former (34.2%) than never (31.2) or current (22.8%) users (Table 2). Severe symptoms were reported by 9.2% (113/1224) of all HCWs. No difference in the severity of symptoms, the likelihood of contact with a confirmed case, or having a positive SARS-CoV-2 test was detected by HCWs’ tobacco/nicotine use status. Among HCWs with positive SARS-CoV-2 test results, 40.4% (109/270) reported symptoms suggestive of COVID-19 at baseline or follow-up with no association of HCWs’ tobacco/nicotine use status with having symptoms or their severity (Table 2). A detailed description of symptoms by HCWs’ tobacco/nicotine use status among the total sample (*n* = 4040) and among HCWs with positive SARS-CoV-2 test results (*n* = 270) is presented in Supplementary Table 2. The hospitalization rate among HCWs was 3.0% (120/4040); no difference was detected by HCWs’ tobacco/nicotine use status (Table 2).

### Factors associated with tobacco/nicotine use status

Factors independently associated with HCWs’ tobacco/nicotine use status are shown in Table 4 using multinomial regression. Compared to never users, former users were more likely to be older [the odds of quitting increased by 5% per year (OR_adjusted_ 1.05, 95%CI: 1.01–1.08, *p* = 0.005)], male (OR_adjusted_ 36.75, 95%CI: 16.28–82.93, *p* < 0.001), self-report a pre-existing medical condition (OR_adjusted_ 1.87, 95%CI: 1.05–3.33, *p* = 0.033), and to experience symptoms (OR_adjusted_ 1.76, 95%CI: 1.07–2.90, *p* = 0.027). Compared to never users, current users were more likely to be male (OR_adjusted_ 72.12, 95%CI: 45.23–114.99, *p* < 0.001) and less educated (OR_adjusted_ 1.42, 95%CI: 1.17–1.71, *p* < 0.001). Compared to physicians, the odds of being a current smoker almost tripled among HCWs involved in non-clinical (OR_adjusted_ 3.21, 95%CI: 2.21–4.67, *p* < 0.001) or nursing occupations (OR_adjusted_ 2.81, 95%CI: 2.01–3.92, *p* < 0.001) (Table 4).

**Table 4:** Factors associated with tobacco/nicotine use among healthcare workers (n = 4040)

	Tobacco/nicotine use			
	Former user		Current user	
	Adjusted OR (95% CI)^a^	*p*-value	Adjusted OR (95% CI)^a^	*p*-value
**Age** (increasing)	1.05 (1.01–1.08)	0.005	1.00 (0.99–1.02)	0.614
**Gender** (male)	36.75 (16.28–82.93)	< 0.001	72.12 (45.23–114.99)	< 0.001
**Residence** (Cairo)	1.29 (0.62–2.68)	0.493	1.26 (0.90–1.76)	0.174
**Area** (Urban)	1.10 (0.47–2.58)	0.832	0.91 (0.62–1.33)	0.619
**Marital status** (married)	0.71 (0.39–1.27)	0.246	1.08 (0.81–1.44)	0.596
**Education** (lower)	1.23 (0.83–1.83)	0.296	1.42 (1.17–1.71)	< 0.001
**Occupation** (nurse)^b^	1.21 (0.61–2.40)	0.596	2.81 (2.01–3.92)	< 0.001
**Occupation** (non-clinical)^b^	1.29 (0.59–2.82)	0.529	3.21 (2.21–4.67)	< 0.001
**Pre-existing medical condition** (yes)	1.87 (1.05–3.33)	0.033	1.22 (0.86–1.74)	0.271
**Symptoms** (yes)	1.76 (1.07–2.90)	0.027	1.07 (0.82–1.40)	0.619
**SARS-Co-V2 test result** (positive)	0.74 (0.26–2.09)	0.575	0.68 (0.41–1.13)	0.136

^a^ Multinomial logistic regression. “Never smoker” was the reference category

^b^ Reference category was physicians

Variables included in the model were age, gender, marital status, governorate and area of residence, level of education, occupation, pre-existing medical conditions, presence of symptoms, and SARS-Co-V2 test result

### Factors associated with positive SARS-CoV-2 test results

Bivariate analysis of factors associated with HCWs’ SARS-CoV-2 test results is shown in Table 5. After adjusting for other variables in the multivariable regression analysis, former (OR_adjusted_ 0.45, 95%CI: 0.11–1.89, *p* = 0.273) and current (OR_adjusted_ 0.65, 95%CI: 0.38–1.09, *p* = 0.101) tobacco/nicotine use was not associated with HCWs’ SARS-CoV-2 positive test results. Older age [compared to the 18–24 years age group: 30–39 years (OR_adjusted_ 1.89, 95%CI: 1.09–3.25, *p* = 0.022), 40–49 years (OR_adjusted_ 1.95, 95%CI: 1.10–3.48, *p* = 0.023)], lower level of education [compared to university/higher: secondary/equivalent (OR_adjusted_ 1.65, 95%CI: 1.08–2.52, *p* = 0.021), primary/preparatory (OR_adjusted_ 2.08, 95%CI: 1.18–3.65, *p* = 0.011)], and experiencing symptoms (OR_adjusted_ 1.65, 95%CI: 1.25–2.16, *p* < 0.001) were independently associated with HCWs’ positive SARS-CoV-2 test results (Table 6). After excluding electronic device users (*n* = 4006) and running the same regression models, no change in the factors associated with SARS-Co-V-2 test results has occurred (Supplementary Table 3).

**Table 5:** Background characteristics of healthcare workers by SARS-Co-V2 test result (n = 4040)

		SARS-Co-V2 test			
	Total	Negative	Positive	Unadjusted odds ratio^a^	*p*-value^a^
	N = 4040	n = 3770	n = 270		
	n (%)	n (%)	n (%)	(95% CI)	
**Age**					
18–24	603 (14.9)	578 (15.3)	25 (9.3)	Ref	
25–29	1279 (31.7)	1220 (32.4)	56 (21.9)	1.12 (0.69–1.80)	0.647
30–39	1057 (26.2)	976 (25.9)	81 (30.0)	1.92 (1.21–3.04)	0.005
40–49	700 (17.3)	632 (16.8)	68 (25.2)	2.49 (1.55–3.99)	< 0.001
≥ 50	401 (9.9)	364 (9.7)	37 (13.7)	2.35 (1.39–3.97)	0.001
**Gender**					
Male	1554 (38.5)	1470 (37.0)	84 (31.1)	Ref	
Female	2486 (61.5)	2300 (61.0)	186 (68.9)	1.42 (1.08–1.85)	0.010
**Governorate of residence**					
Outside Cairo	978 (24.2)	917 (24.3)	62 (23.0)	Ref	
Cairo	3062 (75.8)	2853 (75.7)	208 (77.0)	1.08 (0.80–1.45)	0.614
**Urban/rural residence**					
Rural	497 (12.3)	464 (12.3)	33 (12.2)	Ref	
Urban	3543 (87.7)	3306 (87.7)	237 (87.8)	1.00 (0.69–1.47)	0.967
**Marital status**					
Not married	1865 (46.2)	1779 (47.2)	86 (31.9)	Ref	
Married	2175 (53.8)	1991 (52.8)	184 (68.1)	1.91 (1.47–2.49)	< 0.001
**Education**					
University/higher	2122 (52.5)	2034 (54.0)	88 (32.6)	Ref	
Secondary/equivalent	1551 (38.4)	1405 (37.3)	146 (54.1)	2.40 (1.83–3.16)	< 0.001
Primary/preparatory	238 (5.9)	213 (5.6)	25 (9.3)	2.71 (1.70–4.32)	< 0.001
Less than primary	129 (3.2)	118 (3.1)	11 (4.1)	2.15 (1.12–4.14)	0.021
**Occupation**					
Physician	1577 (39.0)	1519 (40.3)	58 (21.5)	Ref	
Nurse	1598 (39.6)	1447 (38.4)	151 (55.9)	1.99 (1.37–2.89)	< 0.001
Non-clinical care	865 (21.4)	804 (21.3)	61 (22.6)	2.73 (2.00–3.73)	< 0.001
**Tobacco/nicotine use**					
Never	3482 (86.2)	3239 (85.9)	243 (90.0)	Ref	
Former	79 (1.9)	75 (2.0)	4 (1.5)	0.71 (0.26–1.96)	0.510
Current	479 (11.9)	456 (12.1)	23 (8.5)	0.67 (0.43–1.04)	0.076
**Pre-existing medical condition, yes**	701 (17.4)	638 (16.9)	63 (23.3)	1.49 (1.11–2.01)	0.008
**Contact with a confirmed case, yes**^b^	1558 (38.6)	1446 (38.4)	112 (41.5)	1.14 (0.89–1.46)	0.308
**Symptoms, yes**^b^	1224 (30.3)	1115 (29.6)	109 (40.4)	1.61 (1.25–2.08)	< 0.001
**Symptoms severity**	**n = 1224**	***n*** **= 1115**	**n = 109**		
Mild	652 (53.3)	612 (54.9)	40 (36.7)	Ref	
Moderate	459 (37.5)	404 (36.2)	55 (50.5)	2.08 (1.36–3.19)	0.001
Severe	113 (9.2)	99 (8.9)	14 (12.8)	2.16 (1.14–4.12)	0.019

^a^ Bivariable logistic regression analysis

^b^ Numbers indicate any occurrence at either baseline or follow-up phases

**Table 6:** Factors associated with positive SARS-Co-V2 test among healthcare workers (n = 4040)

	Total positive SARS-Co-V2 test at baseline and follow-up(N=270)		Positive SARS-Co-V2 test at baseline (N=170)		Positive SARS-Co-V2 test at follow-up(N=100)	
	Adjusted odds ratio^a^	*p*-value	Adjusted odds ratio^a^	*p*-value	Adjusted odds ratio^a^	*p*-value
	(95% CI)		(95% CI)		(95% CI)	
**Age**						
18–24	Ref		Ref		Ref	
25–29	1.47 (0.87–2.47)	0.147	0.85 (0.37–1.94)	0.700	0.34 (0.099–1.18)	0.090
30–39	1.89 (1.09–3.25)	0.022	1.18 (0.58–2.39)	0.647	0.40 (0.16–0.97)	0.041
40–49	1.95 (1.10–3.48)	0.023	1.24 (0.65–2.34)	0.516	0.95 (0.52–1.75)	0.879
≥ 50	1.88 (0.99–3.57)	0.052	1.44 (0.76–2.73)	0.258	0.84 (0.47–1.52)	0.563
**Gender**						
Male	Ref		Ref		Ref	
Female	0.92 (0.66–1.27)	0.595	0.70 (0.48–1.02)	0.066	1.43 (0.76–2.67)	0.267
**Marital status**						
Not married	Ref		Ref		Ref	
Married	1.08 (0.76–1.54)	0.662	1.04 (0.67–1.61)	0.857	1.25 (0.68–2.29)	0.475
**Education**						
University/higher	Ref		Ref		Ref	
Secondary/equivalent	1.65 (1.08–2.52)	0.021	1.56 (0.92–2.64)	0.097	1.97 (0.99–3.94)	0.055
Primary/preparatory	2.08 (1.18–3.65)	0.011	1.73 (0.81–3.67)	0.156	2.84 (1.23–6.53)	0.014
Less than primary	1.72 (0.81–3.67)	0.159	1.06 (0.30–3.83)	0.926	2.75 (1.02–7.40)	0.045
**Occupation**						
Physician	Ref		Ref		Ref	
Nurse	1.58 (0.95–2.63)	0.077	1.66 (0.90–3.07)	0.106	1.01 (0.40–2.55)	0.988
Non-clinical care	1.25 (0.76–2.07)	0.385	0.99 (0.52–1.87)	0.976	1.21 (0.50–2.93)	0.665
**Tobacco/nicotine use**						
Never	Ref		Ref		Ref	
Former	0.45 (0.11–1.89)	0.273	0.27 (0.04–2.02)	0.202	1.02 (0.13–8.08)	0.985
Current	0.65 (0.38–1.09)	0.101	0.55 (0.29–1.04)	0.066	0.92 (0.38–2.26)	0.857
**Pre-existing medical condition**						
No	Ref		Ref		Ref	
Yes	1.17 (0.84–1.62)	0.354	0.87 (0.56–1.35)	0.531	1.46 (0.90–2.37)	0.124
**Contact with a confirmed case**						
No	Ref		Ref		Ref	
Yes	0.99 (0.76–1.30)	0.990	1.49 (1.06–2.08)	0.021	0.68 (0.44–1.06)	0.087
**Symptoms**						
No	Ref		Ref		Ref	
Yes	1.65 (1.25–2.16)	< 0.001	2.59 (1.81–3.72)	< 0.001	1.59 (1.01–2.50)	0.047

^a^ Multivariable logistic regression analysis

For the three models, variables included were age, gender, marital status, education, occupation, pre-existing medical condition, contact with a confirmed case, symptoms, tobacco use, and tobacco use-pre-existing medical conditions interaction term. For the interaction term adjusted OR at both baseline and follow-up phases: in former users = 2.84, 95% CI: 0.35–22.94, *p* = 0.327; and in current users = 0.52, 95% CI: 0.11–2.35, *p* = 0.393

## Discussion

In the studied cohort of HCWs, having a positive SARS-CoV-2 test was not associated with current, former, or never tobacco/nicotine use after accounting for demographics, exposure history, and presence of symptoms and co-morbidities. Unlike current users, former users were significantly more likely to have co-morbidities and to report symptoms than never users, however, with no difference in their severity. Among HCWs with positive SARS-CoV-2 test results, the presence of symptoms or their severity did not vary by tobacco/nicotine use status. The current tobacco/nicotine use profile was not significantly different among SARS-CoV-2 positive and negative HCWs.

Our data did not provide evidence supportive of the hypothesized protective effect of smoking against COVID-19 that was discussed in previous studies [3, 21]. The relation between nicotine use and SARS-CoV-2 infection is not fully understood. It was postulated that SARS-CoV-2 uses angiotensin converting enzyme 2 receptors (ACE-2) to enter host cells [22]. Some studies suggest that current smokers showed an elevated expression of ACE-2 receptors [23], which might increase their susceptibility for SARS-CoV-2 infection. However, other studies suggest that nicotine acts through downregulating ACE-2 receptor expression [24]. Therefore, the mechanistic association between nicotine and SARS-CoV-2 infection merits additional investigation. Evidence of protective effect was mostly derived from studies among hospitalized patients with data taken from health records or from small convenient hospital samples that lack representation of those short to reach healthcare [3, 21]. This apparent protective effect should be taken cautiously because of the potential bias of self-selection of infected smokers; being less likely to seek medical care or to receive testing due to their inability to pay [25], or due to experiencing severe and fatal complications before hospitalization [3]. Underreporting of current smoking and the misclassification of former smokers as never smokers are other sources of information bias in hospital-based studies [26]. Also depending on hospital records, smokers who suffered severe symptoms that warranted hospitalization may quit just before admission, which inflates the risk among former smokers if they were correctly classified as former smokers, and among never smokers if incorrectly classified as never smokers [3]. Therefore, obtaining complete tobacco use history with more accurate time of quitting could help resolve this information bias within hospital records.

On the other hand, based on this analysis and our previous findings [15], HCWs’ data did not support a higher risk of SARS-CO-V2 infection among current tobacco users. RT-PCR test sensitivity was found to be lower among current smokers due to the effect of smoking on the nasopharyngeal viral load [27]. This may have resulted in reduced case detection and consequently masked a hypothesized association. Also, compared to never users, we did not find evidence for a higher odds of reporting symptoms suggestive of COVID-19 by current tobacco/nicotine users. By contrast, current smoking in a large population-based cohort (2.4 million) increased the risk of reporting symptoms suggestive of COVID-19, and among infected smokers it increased the symptom burden and the need for hospital attendance [7]. In this HCW cohort, only former tobacco/nicotine users were significantly more likely to report symptoms suggestive of COVID-19 (OR_adjusted_ 1.76, 95%CI: 1.07–2.90, *p* = 0.027) and also to report pre-existing comorbidities (OR_adjusted_ 1.87, 95%CI: 1.05–3.33, *p* = 0.033); particularly obesity, cardiovascular disease, and COPD. The latter finding could be explained by reverse causation; having comorbidities led them to quit smoking for their sense of vulnerability to the higher or actual occurrence of disease complications or adverse outcomes. This state of health consciousness may suggest more reporting of symptoms by former users than never or current users. Jackson et al. found that smokers had quit due to experiencing symptoms [6]. However, most HCWs (62/79, 78.5%) had quit between 2 months to ≥1 year before the study was conducted (i.e., before reporting of the first confirmed case in Egypt on mid-February) and there was no significant association between the time of quitting and experiencing symptoms (Table 3). Therefore, quitting due to symptoms suggestive of COVID-19 does not explain the higher odds of symptoms among former users in this HCW cohort. Noteworthy, health warnings about the association of tobacco use with COVID-19 was not a common reason for quitting, 26.6% (21/79) among former users in general. Although 75.0% (3/4) of former users who were SARS-CoV-2 positive reported quitting due to these health warnings, the difference did not vary significantly by test results probably due to insufficient numbers.

Among SARS-CoV-2 positive HCWs, the absence of a significant difference in the presence of symptoms or their severity by tobacco/nicotine use status is probably inconclusive—owing to the insufficient number of symptomatic positive HCWs who were current (*n* = 7) and former users (*n* = 2). Also, we detected no difference in the proportion of hospitalization by tobacco/nicotine use status (Table 2). According to ASU hospital policy at that early phase of the pandemic in Egypt, cases may have been hospitalized for isolation purposes rather than due to experiencing severe disease.

Our findings are in line with those from a large UK population-based study (53,002 participants), which reported there was no association of COVID-19 among participants who attained higher education with post-16 qualification [6]. Jackson et al. reported 1.8 times higher odds of confirmed COVID-19 among current smokers relative to never smokers; this association was manifested only among the less educated group (no post-16 qualifications). An important uniqueness to our study sample of HCWs is that most (90.9%) had attained higher education (university/higher = 52.5%, secondary/equivalent = 38.4%). In this HCW cohort, the effect of tobacco/nicotine use on SARS-CoV-2 test results did not differ across educational levels given the insignificance of the interaction term between the level of education and tobacco/nicotine use (OR 0.91 95%CI: 0.76–1.08, *p* = 0.271) (data not tabulated). These sample characteristics could make our finding of absence of association comparable with those of the post-16 qualification group in UK study [6].

### Strengths and limitations

This cohort study design enabled the identification of recent occurrence of symptoms, exposures, and infections in HCWs, specifically in that early phase of the epidemic in Egypt. HCW screening was universal with no preset criteria for SARS-CoV-2 testing. In line with previous calls for study analyses to account for the potential confounders that may affect the association between participants’ smoking and SARS-CoV-2 positivity, our analysis adjusted for demographic characteristics, exposures, and pre-existing co-morbidities, disentangling the specific effect of smoking.

This HCW cohort included a higher proportion of more educated individuals and a lower proportion of chronic diseases and current/former tobacco use than in the general Egyptian population (90.9% versus 42.4, 17.4% versus ~ 23, and 11.9%/1.9% versus 22.7%/3.2%, respectively) [12]. However, these characteristics most probably did not bias the association measured because the comparison groups were from the same cohort and potential confounders were accounted for during the analysis; thus, internal validity was likely preserved. Despite being a special group, this HCW cohort study is unlikely to have collider bias that may inflate the association in hospital-based studies [28]. Also, misclassification of the exposure is unlikely as data on tobacco/nicotine use status (never, former, and current) were collected in detail.

Our results did not provide evidence supportive of a higher odds of reporting symptoms suggestive of COVID-19 by current than never users. A recall bias may have distorted this association; unlike never users, current users usually suffer symptoms such as morning cough and expectoration, change/loss of taste and smell, shortness of breath, wheezy chest, etc., which they might have considered as their norm. Hence, reporting of such symptoms, particularly when their severity is mildly changed, might have been overlooked by current users but were not likely to be overlooked by never users. Also, the limited number of symptomatic SARS-CoV-2 positive HCWs that were current or former users may have led to insufficient power to obtain conclusive results about the effect of tobacco/nicotine use on the occurrence of symptoms or their severity.

This study was conducted amid the peak of the first wave of COVID-19 epidemic in Egypt. Some HCWs were quarantined or self-isolating and others were redistributed to cover shortages in HCW staff in the newly established COVID-19 isolation wards/hospitals. Thus, many HCWs were not able to attend their follow-up appointments. Although this longitudinal study had somewhat a high proportion of lost to follow-up (~ 40%), the proportions of HCWs within each category of tobacco/nicotine use did not differ between baseline and follow-up phases (recapturing of symptoms, exposures, and testing) indicating the absence of a biased distribution of tobacco/nicotine use status between the two phases (Table 1).

Although there is a distinction between combustible tobacco and electronic nicotine delivery products as regards their toxicant and nicotine contents, findings of no association with SARS-CoV-2 positivity were alike after running separate regression analyses for tobacco/nicotine users and for tobacco only users in this cohort of HCWs. Future investigations could examine whether there is an association of nicotine use with SARS-CoV-2 infection risk in more detail and among a larger number of electronic device users.

## Conclusion

Among this HCW cohort, there was no association of tobacco/nicotine use with SARS-CoV-2 positivity in current or former users compared with never users—after accounting for demographics, exposure history, and presence of symptoms and co-morbidities. Former users were significantly more likely to have self-reported pre-existing medical conditions and to experience symptoms than never users. The presence of symptoms and the severity thereof did not vary by tobacco/nicotine use status in HCWs with positive SARS-CoV-2 test results. Additional studies with population-level representative samples are warranted to study this association in more depth, especially in countries with a high prevalence of smoking among the general population like the case in Egypt.

## Supplementary Information

**Additional file 1: Supplementary Table 1**. Self-reported pre-existing medical conditions among healthcare workers (*n* = 4040). **Supplementary Table 2**. Symptoms reported by healthcare workers (n = 4040). **Supplementary Table 3.** Factors associated with positive SARS-Co-V2 test among healthcare workers excluding electronic device users (*n* = 4006).

## Data Availability

All data generated or analysed during this study are included in this published article and its supplementary information files.

## References

[1] World Health Organization. Noncommunicable diseases. Geneva; 2017. https://www.who.int/news-room/fact-sheets/detail/noncommunicable-diseases. Accessed 20 March 2021.

[2] Sitas F, Harris-Roxas B, Bradshawb D, Lopez AD. Smoking and epidemics of respiratory infections. Bulletin of the World Health Organization. November 2020 https://www.who.int/bulletin/online_first/BLT.20.273052.pdf. Accessed 29 January 2021. 10.2471/BLT.20.273052 PMC7856358 33551511

[3] Simons D, Shahab L, Brown J, Perski O. The association of smoking status with SARS-CoV-2 infection, hospitalization and mortality from COVID-19: a living rapid evidence review with Bayesian meta-analyses (version 7). Addiction. 2020. 10.1111/add.15276. 10.1111/add.15276 PMC7590402 33007104

[4] Forouzanfar MH, Afshin A, Alexander LT, Anderson HR, Bhutta ZA, Biryukov S (2016). GBD 2015 Risk Factors Collaborators. Global, regional, and national comparative risk assessment of 79 behavioural, environmental and occupational, and metabolic risks or clusters of risks, 2015–1990: a systematic analysis for the Global Burden of Disease Study 2015. Lancet.

[5] Farsalinos K, Barbouni A, Poulas K (2020). Current smoking, former smoking, and adverse outcome among hospitalized COVID-19 patients: a systematic review and meta-analysis. Ther Adv Chronic Dis.

[6] Jackson SE, Brown J, Shahab L, Steptoe A, Fancourt D. COVID-19, smoking and inequalities: a study of 53 002 adults in the UK. Tob Control. 2020 Aug 21:tobaccocontrol-2020-055933. doi: 10.1136/tobaccocontrol-2020-055933. 10.1136/tobaccocontrol-2020-055933 32826387

[7] Hopkinson NS, Rossi N, El-Sayed Moustafa J, Laverty AA, Quint JK, Freidin M, et al. Current smoking and COVID-19 risk: results from a population symptom app in over 2.4 million people. Thorax. 2021: thoraxjnl-2020-216422. 10.1136/thoraxjnl-2020-216422. 10.1136/thoraxjnl-2020-216422 33402392

[8] Wenzl T (2020). Smoking and COVID-19: Did we overlook representativeness?. Tob Induc Dis.

[9] Ministry of Health and Population, Egypt. COVID-19 report. https://www.care.gov.eg/EgyptCare/index.aspx. Accessed 18 March 2021.

[10] Adbelkhalik AA, Kamel LM, Afifi ZAM, Radwan GN, El-Khashab SO (2019). Knowledge, Attitude and Practice of University Hospital Staff regarding tobacco control measures. Med J Cairo Univ.

[11] Abou-ElWafa HS, Zoromba MA, El-Gilany AH (2021). Cigarette smoking at workplace among resident physicians and nurses in Mansoura University hospital. Arch Environ Occup Health.

[12] Egypt National STEPwise Survey for Noncommunicable Diseases Risk Factors Report 2017. A joint report by the Egyptian Ministry of Health and Population and the World Health Organization. https://www.who.int/ncds/surveillance/steps/Egypt_National_STEPwise_Survey_For_Noncommunicable_Diseases_Risk_Factors_2017_Report.pdf?ua=1. Accessed 29 January 2021.

[13] Tageldin MA, Nafti S, Khan JA, Nejjari C, Beji M, Mahboub B, Obeidat NM (2012). BREATHE Study Group. Distribution of COPD-related symptoms in the Middle East and North Africa: results of the BREATHE study. Respir Med.

[14] Mostafa A, Kandil S, El-Sayed MH, Girgis S, Hafez H, Yosef M, et al. Universal COVID-19 screening of 4040 health care workers in a resource-limited setting: an Egyptian pilot model in a university with 12 public hospitals and medical centers. Int J Epidemiol. 2020:dyaa173. 10.1093/ije/dyaa173. 10.1093/ije/dyaa173 PMC7665557 33094320

[15] Mostafa A, Kandil S, El-Sayed MH, Girgis S, Hafez H, Yosef M (2021). SARS-CoV-2 seroconversion among 4040 Egyptian healthcare workers in 12 resource-limited healthcare facilities: A prospective cohort study. Int J Infect Dis.

[16] World Health Organization. (2020). Population-based age-stratified seroepidemiological investigation protocol for COVID-19 virus infection, 17 march 2020. World Health Organization. https://apps.who.int/iris/handle/10665/331656. Accessed 09 April 2020.

[17] Lassaunière R, Frische A, Harboe ZB, Nielsen ACY, Fomsgaard A, Krogfelt KA, et al. Evaluation of Nine Commercial SARS-CoV-2 Immunoassays. medRxiv. 2020. 10.1101/2020.04.09.20056325.

[18] Zhang and Zheng Artron Laboratories Inc. R & D Department Quality control department. IgM/IgG Antibody Test Diagnostic Sensitivity and Specificity Study Report May 21, 2020. COVID-19. http://www.cemagcare.com/wpcontent/uploads/2020/07/Rapport_de_performances_test_de_detection_covid19_IgM_IgG_Artr n.pdf. Accessed 31 December 2020.

[19] Centers for Disease Control and Prevention 2020a. Coronavirus Disease 2019 (COVID-19). Laboratories. Resources for Labs. Interim Guidelines for Collecting, Handling, and Testing Clinical Specimens for COVID-19. https://www.cdc.gov/corona virus/2019-ncov/lab/guidelines-clinical-specimens.html. Accessed 09 April 2020.

[20] Van Kasteren PB, van der Veer B, van den Brink S, Wijsman L, de Jonge J, van den Brandt A (2020). Comparison of seven commercial RT-PCR diagnostic kits for COVID-19. J Clin Virol.

[21] Grundy EJ, Suddek T, Filippidis FT, Majeed A, Coronini-Cronberg S (2020). Smoking, SARS-CoV-2 and COVID-19: A review of reviews considering implications for public health policy and practice. Tob Induc Dis.

[22] Hoffmann M, Kleine-Weber H, Schroeder S, Krüger N, Herrler T, Erichsen S, Schiergens TS, Herrler G, Wu NH, Nitsche A, Müller MA, Drosten C, Pöhlmann S (2020). SARS-CoV-2 cell entry depends on ACE2 and TMPRSS2 and is blocked by a clinically proven protease inhibitor. Cell.

[23] Brake SJ, Brake SJ, Barnsley K, Lu W, McAlinden KD, Eapen MS, Sohal SS (2020). Smoking Upregulates Angiotensin-Converting Enzyme-2 Receptor: A Potential Adhesion Site for Novel Coronavirus SARS-CoV-2 (Covid-19). J Clin Med.

[24] Oakes JM, Fuchs RM, Gardner JD, Lazartigues E, Yue X (2018). Nicotine and the renin-angiotensin system. Am J Phys Regul Integr Comp Phys.

[25] Hiscock R, Bauld L, Amos A, Amos A, Fidler J, Munafò M (2012). Socioeconomic status and smoking: a review. Ann N Y Acad Sci.

[26] Polubriaginof F, Salmasian H, Albert DA, Vawdrey DK (2018). Challenges with Collecting Smoking Status in Electronic Health Records. AMIA Annu Symp Proc.

[27] de Lusignan S, Dorward J, Correa A, Jones N, Akinyemi O, Amirthalingam G, Andrews N, Byford R, Dabrera G, Elliot A, Ellis J, Ferreira F, Lopez Bernal J, Okusi C, Ramsay M, Sherlock J, Smith G, Williams J, Howsam G, Zambon M, Joy M, Hobbs FDR (2020). Risk factors for SARS-CoV-2 among patients in the Oxford Royal College of general practitioners research and surveillance Centre primary care network: a cross-sectional study. Lancet Infect Dis.

[28] Griffith G, Morris TT, Tudball M, Herbert A, Mancano G, Pike L, et al. Collider bias undermines our understanding of COVID-19 disease risk and severity. medRxiv. 2020. 10.1101/2020.05.04.20090506. 10.1038/s41467-020-19478-2 PMC7665028 33184277

