# Supplementary material for: SARS-Co-V2 infection in never, former, and current tobacco/nicotine users: a cohort study of 4040 Egyptian healthcare workers
Source: BMC Public Health. 2021 Jun 28;21:1243. doi: 10.1186/s12889-021-11290-x (PMC8238378; doi:10.1186/s12889-021-11290-x)
Supplement: Supplementary file 1 — Additional file 1: Supplementary Table 1. Self-reported pre-existing medical conditions among healthcare workers (n = 4040). Supplementary Table 2. Symptoms reported by healthcare workers (n = 4040). Supplementary Table 3. Factors associated with positive SARS-Co-V2 test among healthcare workers excluding electronic device users (n = 4006). [file 12889_2021_11290_MOESM1_ESM.docx]

**Supplementary Table 1. Self-reported pre-existing medical conditions among healthcare workers (n=4040)**

|  | **Total** | **Tobacco/nicotine use in the total sample** | | | | **Positive test** | **Proportion of infection** | **Tobacco/nicotine use in HCW with positive tests** | | | |
| --- | --- | --- | --- | --- | --- | --- | --- | --- | --- | --- | --- |
|  |  | **Never** | **Former** | **Current** | **p-value*** |  |  | **Never** | **Former** | **Current** | **p-value*** |
|  | **N=4040** | **n=3482** | **n=79** | **n=479** |  | **n=270** |  | **n=243** | **n=4** | **n=23** |  |
|  | **n (%)** | **n (%)** | **n (%)** | **n (%)** |  | **n (%)** | **row%(95%CI)** | **n (%)** | **n (%)** | **n (%)** |  |
| **Pre-existing medical condition(s)**** | | | | | | | | | | | |
| Hypertension | 291 (7.2) | 261 (7.5) | 6 (7.6) | 24 (5.0) | 0.142 | 28 (10.4) | 9.6 (6.2-13.0) | 27 (11.1) | 0 | 1 (4.3) | 0.669 |
| Diabetes | 176 (4.4) | 153 (4.4) | 2 (2.5) | 21 (4.4) | 0.725 | 15 (5.6) | 8.5 (4.4-12.7) | 15 (6.2) | 0 | 0 | 0.505 |
| Obesity | 145 (3.6) | 120 (3.4) | 8 (10.1) | 17 (3.5) | 0.007 | 9 (3.3) | 6.2 (2.2-10.2) | 8 (3.3) | 1 (25.0) | 0 | 0.157 |
| COPD (needs medication) | 68 (1.7) | 63 (1.8) | 1 (1.3) | 4 (0.8) | 0.287 | 4 (1.5) | 5.9 (0.1-11.6) | 3 (1.2) | 1 (25.0) | 0 | 0.095 |
| COPD (does not need medication) | 27 (0.7) | 21 (0.6) | 4 (5.1) | 2 (0.4) | <0.001 | 0 | 0 | 0 | 0 | 0 | na |
| Cardiovascular disease | 55 (1.4) | 49 (1.4) | 5 (6.3) | 1 (0.2) | <0.001 | 5 (1.9) | 9.1 (1.3-16.9) | 5 (2.1) | 0 | 0 | 1.000 |
| Immunological disorder | 36 (0.9) | 36 (1.0) | 0 | 0 | 0.054 | 6 (2.2) | 16.7 (3.9-29.5) | 6 (2.5) | 0 | 0 | 1.000 |
| Pregnancy | 33 (0.8) | 33 (0.9) | 0 | 0 | 0.070 | 4 (1.5) | 12.1 (0.4-23.9) | 4 (1.6) | 0 | 0 | 1.000 |
| Chronic kidney disease | 13 (0.3) | 11 (0.3) | 0 | 2 (0.4) | 0.820 | 3 (1.1) | 23.1 (-3.4-49.6) | 3 (1.2) | 0 | 0 | 1.000 |
| Chronic liver disease | 9 (0.2) | 8 (0.2) | 1 (1.3) | 0 | 0.084 | 1 (0.4) | 11.1 (-14.5-36.7) | 1 (0.4) | 0 | 0 | 1.000 |
| Neurological disorder | 8 (0.2) | 6 (0.2) | 1 (1.3) | 1 (0.2) | 0.096 | 0 | 0 | 0 | 0 | 0 | na |
| Neoplasm | 8 (0.2) | 8 (0.2) | 0 | 0 | 0.526 | 0 | 0 | 0 | 0 | 0 | na |
| Chronic hematological disease | 6 (0.1) | 5 (0.1) | 0 | 1 (0.2) | 0.887 | 1 (0.4) | 16.7 (-26.2-59.5) | 0 | 0 | 1 (4.3) | 0.100 |

*Chi-squared test or Fisher's Exact test

** Numbers/% do not sum to total number/100% because more than one option is possible

**Supplementary Table 2. Symptoms reported by healthcare workers (n=4040)**

|  | **Total** | **Tobacco/nicotine use in the total sample** | | | | **Positive test** | **Proportion of infection** | **Tobacco/nicotine use in HCW with positive tests** | | | |
| --- | --- | --- | --- | --- | --- | --- | --- | --- | --- | --- | --- |
|  |  | **Never** | **Former** | **Current** | **p-value*** |  |  | **Never** | **Former** | **Current** | **p-value*** |
|  | **N=4040** | **n=3482** | **n=79** | **n=479** |  | **n=270** |  | **n=243** | **n=4** | **n=23** |  |
|  | **n (%)** | **n (%)** | **n (%)** | **n (%)** |  | **n (%)** | **row%(95%CI)** | **n (%)** | **n (%)** | **n (%)** |  |
| **Symptoms**** | | | | | | | | | | | |
| Fever <38°C | 189 (4.7) | 163 (4.7) | 4 (5.1) | 22 (4.6) | 0.983 | 26 (9.6) | 13.8 (8.8-18.7) | 25 (10.3) | 0 | 1 (4.3) | 0.807 |
| Fever ≥38°C | 82 (2.0) | 72 (2.1) | 3 (3.8) | 7 (1.5) | 0.360 | 11 (4.1) | 13.4 (5.9-20.9) | 9 (3.7) | 1 (25.0) | 1 (4.3) | 0.146 |
| Chills | 49 (1.2) | 40 (1.1) | 3 (3.8) | 6 (1.3) | 0.104 | 4 (1.5) | 8.2 (2.2-16.1) | 3 (1.2) | 1 (25.0) | 0 | 0.095 |
| Muscle pain | 234 (5.8) | 211 (6.1) | 3 (3.8) | 20 (4.2) | 0.189 | 27 (10.0) | 11.5 (7.4-15.7) | 24 (9.9) | 1 (25.0) | 2 (8.7) | 0.521 |
| Joint ache | 176 (4.4) | 163 (4.7) | 1 (1.3) | 12 (2.5) | 0.036 | 25 (9.3) | 14.2 (9.0-19.4) | 23 (9.5) | 0 | 2 (8.7) | 1.000 |
| Sore throat | 564 (14.0) | 500 (14.4) | 15 (19.0) | 49 (10.2) | 0.022 | 44 (16.3) | 7.8 (5.6-10.0) | 40 (16.5) | 1 (25.0) | 3 (13.0) | 0.782 |
| Dry cough | 261 (6.5) | 240 (6.9) | 5 (6.3) | 16 (3.3) | 0.012 | 32 (11.9) | 12.3 (8.3-16.3) | 30 (12.3) | 0 | 2 (8.7) | 1.000 |
| Cough with sputum | 158 (3.9) | 131 (3.8) | 4 (5.1) | 23 (4.8) | 0.473 | 17 (6.3) | 10.8 (5.9-15.6) | 15 (6.2) | 0 | 2 (8.7) | 0.729 |
| Runny nose/nasal congestion | 286 (7.1) | 257 (7.4) | 7 (8.9) | 22 (4.6) | 0.068 | 28 (10.4) | 9.8 (6.3-13.3) | 27 (11.1) | 0 | 1 (4.3) | 0.669 |
| Sneezing | 314 (7.8) | 284 (8.2) | 6 (7.6) | 24 (5.0) | 0.055 | 32 (11.9) | 10.2 (6.8-13.6) | 31 (12.8) | 0 | 1 (4.3) | 0.596 |
| Shortness of breath | 142 (3.5) | 124 (3.6) | 4 (5.1) | 14 (2.9) | 0.584 | 17 (6.3) | 11.9 (6.6-17.4) | 14 (5.8) | 0 | 3 (13.0) | 0.363 |
| Wheezing | 26 (0.6) | 21 (0.6) | 0 | 5 (1.0) | 0.397 | 1 (0.4) | 3.9 (-4.1-11.8) | 1 (0.4) | 0 | 0 | 1.000 |
| Chest pain | 56 (1.4) | 49 (1.4) | 1 (1.3) | 6 (1.3) | 0.960 | 9 (3.3) | 16.1 (6.2-26.0) | 8 (3.3) | 0 | 1 (4.3) | 0.618 |
| Other respiratory symptoms | 23 (0.6) | 21 (0.6) | 1 (1.3) | 1 (0.2) | 0.286 | 6 (2.2) | 26.1 (6.7-45.5) | 6 (2.5) | 0 | 0 | 1.000 |
| Nausea | 49 (1.2) | 46 (1.3) | 1 (1.3) | 2 (0.4) | 0.238 | 3 (1.1) | 6.1 (-0.8-13.1) | 3 (1.2) | 0 | 0 | 1.000 |
| Vomiting | 27 (0.7) | 26 (0.7) | 0 | 1 (0.2) | 0.727 | 3 (1.1) | 11.1 (-1.6-23.8) | 3 (1.2) | 0 | 0 | 1.000 |
| Abdominal discomfort/pain | 94 (2.3) | 83 (2.4) | 2 (2.5) | 9 (1.9) | 0.784 | 10 (3.7) | 10.6 (4.3-16.9) | 9 (3.7) | 0 | 1 (4.3) | 0.658 |
| Diarrhea | 184 (4.6) | 164 (4.7) | 6 (7.6) | 14 (2.9) | 0.090 | 21 (7.8) | 11.4 (6.8-16.1) | 19 (7.8) | 1 (25.0) | 1 (4.3) | 0.346 |
| Headache | 310 (7.7) | 270 (7.8) | 7 (8.9) | 33 (6.9) | 0.739 | 39 (14.4) | 12.6 (8.9-16.3) | 36 (14.8) | 0 | 3 (13.0) | 1.000 |
| Dizziness | 50 (1.2) | 44 (1.3) | 1 (1.3) | 5 (1.0) | 0.920 | 2 (0.7) | 4.0 (-1.6-9.6) | 1 (0.4) | 0 | 1 (4.3) | 0.190 |
| Confusion | 10 (0.2) | 10 (0.3) | 0 | 0 | 0.689 | 2 (0.7) | 20.0 (-10.6-50.2) | 2 (0.8) | 0 | 0 | 1.000 |
| Loss of appetite | 61 (1.5) | 50 (1.4) | 2 (2.5) | 9 (1.9) | 0.571 | 11 (4.1) | 18.0 (8.1-27.9) | 9 (3.7) | 0 | 2 (8.7) | 0.360 |
| Change/loss of taste | 45 (1.1) | 38 (1.1) | 1 (1.3) | 6 (1.3) | 0.943 | 13 (4.8) | 28.9 (15.1-42.7) | 11 (4.5) | 0 | 2 (8.7) | 0.436 |
| Change/loss of smell | 47 (1.2) | 41 (1.2) | 1 (1.3) | 5 (1.0) | 0.964 | 16 (5.9) | 34.0 (19.9-48.1) | 14 (5.8 ) | 0 | 2 (8.7) | 0.716 |
| Skin rash | 11 (0.3) | 10 (0.3) | 0 | 1 (0.2) | 1.000 | 0 | 0 | 0 | 0 | 0 | na |
| Conjunctivitis | 39 (1.0) | 33 (0.9) | 2 (2.5) | 4 (0.8) | 0.346 | 6 (2.2) | 15.4 (3.5-27.2) | 5 (2.1) | 0 | 1 (4.3) | 0.472 |

*Chi-squared test or Fisher's Exact test

** Numbers/% do not sum to total number/100% because more than one option is possible

**Supplementary Table 3. Factors associated with positive SARS-Co-V2 test among healthcare workers excluding electronic device users (n=4006)**

|  | **Total positive SARS-Co-V2 test at baseline and follow-up (N=269)** | | **Positive SARS-Co-V2 test at baseline  (N=169)** | | **Positive SARS-Co-V2 test at follow-up (N=100)** | |  |
| --- | --- | --- | --- | --- | --- | --- | --- |
|  | **Adjusted  odds ratio^a^** | **p-value** | **Adjusted  odds ratio^a^** | **p-value** | **Adjusted  odds ratio^a^** | **p-value** |  |
|  |  |  |  |  |  |  |  |
|  | **(95% CI)** |  | **(95% CI)** |  | **(95% CI)** |  |  |
| **Age** | | | | | | |  |
| 18-24 | Ref |  | Ref |  | Ref |  |  |
| 25-29 | 1.45 (0.86-2.45) | 0.160 | 1.36 (0.76-2.44) | 0.305 | 1.17 (0.36-3.84) | 0.796 |  |
| 30-39 | 1.90 (1.10-3.27) | 0.021 | 1.46 (0.77-2.76) | 0.248 | 2.78 (0.88-8.80) | 0.082 |  |
| 40-49 | 1.97 (1.11-3.50) | 0.021 | 1.70 (0.86-3.37) | 0.127 | 2.45 (0.74-8.07) | 0.142 |  |
| ≥50 | 1.90 (1.00-3.59) | 0.049 | 1.18 (0.52-2.69) | 0.691 | 2.92 (0.85-10.06) | 0.090 |  |
| **Gender** | | | | | | |  |
| Male | Ref |  | Ref |  | Ref |  |  |
| Female | 0.92 (0.66-1.27) | 0.660 | 0.70 (0.48-1.03) | 0.068 | 1.42 (0.76-2.65) | 0.273 |  |
| **Marital status** |  |  |  |  |  |  |  |
| Not married | Ref |  | Ref |  | Ref |  |  |
| Married | 1.06 (0.74-1.51) | 0.759 | 1.01 (0.65-1.57) | 0.965 | 1.24 (0.68-2.27) | 0.489 |  |
| **Education** | | | | | | |  |
| University/higher | Ref |  | Ref |  | Ref |  |  |
| Secondary/equivalent | 1.64 (1.07-2.51) | 0.023 | 1.55 (0.92-2.62) | 0.103 | 1.96 (0.98-3.92) | 0.056 |  |
| Primary/preparatory | 2.08 (1.18-3.65) | 0.011 | 1.72 (0.81-3.66) | 0.159 | 2.77 (1.20-6.37) | 0.017 |  |
| Less than primary | 1.72 (0.81-3.66) | 0.161 | 1.06 (0.29-3.82) | 0.931 | 2.67 (1.00-7.20) | 0.051 |  |
| **Occupation** | | | | | | |  |
| Physician | Ref |  | Ref |  | Ref |  |  |
| Nurse | 1.58 (0.95-2.63) | 0.079 | 1.66 (0.90-3.06) | 0.108 | 1.01 (0.40-2.56) | 0.984 |  |
| Non-clinical care | 1.24 (0.75-2.06) | 0.396 | 0.98 (0.52-1.86) | 0.961 | 1.22 (0.51-2.95) | 0.657 |  |
| **Tobacco/nicotine use** | | | | | | |  |
| Never | Ref |  | Ref |  | Ref |  |  |
| Former | 0.45 (0.11-1.89) | 0.274 | 0.27 (0.04-2.02) | 0.202 | 1.02 (0.13-8.04) | 0.988 |  |
| Current | 0.66 (0.39-1.13) | 0.127 | 0.57 (0.29-1.10) | 0.093 | 0.97 (0.39-2.37) | 0.939 |  |
| **Pre-existing medical condition** | | | | | | |  |
| No | Ref |  | Ref |  | Ref |  |  |
| Yes | 1.17 (0.84-1.62) | 0.349 | 0.87 (0.56-1.35) | 0.540 | 1.45 (0.89-2.35) | 0.135 |  |
| **Contact with a confirmed case** | | | | | | |  |
| No | Ref |  | Ref |  | Ref |  |  |
| Yes | 0.99 (0.75-1.29) | 0.925 | 1.49 (1.06-2.08) | 0.022 | 0.77 (0.57-1.04) | 0.086 |  |
| **Symptoms** | | | | | | |  |
| No | Ref |  | Ref |  | Ref |  |  |
| Yes | 1.64 (1.25-2.15) | <0.001 | 2.57 (1.79-3.70) | <0.001 | 1.64 (1.03-2.60) | 0.036 |  |

**^a^** Multivariable logistic regression analysis

For the three models, variables included were age, gender, marital status, education, occupation, pre-existing medical condition, contact with a confirmed case, symptoms, tobacco use, and tobacco use-pre-existing medical conditions interaction term. For the interaction term adjusted OR at both baseline and follow-up phases: in former users=2.83, 95% CI: 0.35-22.84, p=0.329; and in current users=0.53, 95% CI: 0.12-2.44, p=0.417.
